# Left atrial mass found on cardiac ultrasound leading to emergent thrombectomy

**DOI:** 10.1016/j.rmcr.2022.101800

**Published:** 2022-12-19

**Authors:** Nathalie van der Rijst, Tejas Sinha, Parth Rali, Parag Desai, Sebastian Iturra, Aamir Ajmeri

**Affiliations:** Temple University Hospital, Department of Thoracic Medicine and Surgery, Philadelphia, PA, USA

## Abstract

Atrial myxomas, though the most common primary cardiac neoplasm, remain a rare disease occurring in about 0.03% of the population. While clinically benign, they are considered functionally malignant as they can cause life-threatening embolic events. Here we present a patient with a high-risk intermediate pulmonary embolism where bedside ultrasound revealed significant right ventricular dysfunction with an associated large left atrial mass. These findings combined with the patient's instability allowed her to be rushed to surgery for definitive treatment.

## 1 Background

Atrial Myxoma is the most common primary cardiac neoplasm and can lead to life-threatening embolic events, including pulmonary embolisms. Pulmonary emboli (PE) remains a leading cause of cardiovascular morbidity and mortality, and risk stratification in acute PE is crucial in deciding management and outcome [1]. Initial risk stratification is based on assessment of hemodynamic status; however, the large majority of PE remain hemodynamically stable with a variable spectrum of severity ranging from small, asymptomatic (i.e., low-risk PE) to those with borderline hypotension or imminent signs of circulatory collapse (i.e., intermediate-risk PE). Thus, optimizing risk stratification of patients with normotensive PE is crucial to allow early detection of hemodynamic decompensation, and the need for advanced reperfusion therapy accordingly. Bedside cardiac ultrasound has been found to be a useful tool in risk stratification by identifying right ventricular (RV) dysfunction and subsequently treatment choice. Cardiac ultrasound is also the diagnostic modality for atrial myxoma [3]. We present a case where cardiac ultrasound for PE risk stratification allowed for the diagnosis of an atrial myxoma with subsequent definitive treatment being offered in the Operating Room (OR)

## 2 Case report

A 68-year-old female was brought to the emergency room by her daughter due to complaints of an ongoing cough for the last 3 weeks now with acute worsening and a change in mental status. The patient had a past medical history of paroxysmal atrial fibrillation and a history of deep venous thromboembolism (DVT), not currently on anticoagulation, as well as Chronic Obstructive Pulmonary Disease (COPD) on home 2L/min supplemental oxygen, obstructive sleep apnea (OSA) on nocturnal BiPAP, heart failure with reduced ejection fraction, WHO Group 3 pulmonary hypertension and a history of cirrhosis of unclear etiology. The patient herself was too altered to provide her own history, and history was obtained from the daughter. As per the patient's daughter, the patient had been increasingly complaining of a cough as well as weakness for the past three weeks. However, the morning of presentation patient became altered and was unable to talk.

Upon initial examination, the patient was afebrile with a blood pressure of 162/104 mmHg, a heart rate of 80bpm and she was saturating 96% on her home 2 L of oxygen. Physical examination was pertinent for confusion and only being oriented to name, an ecchymosis on her left eye, decreased breath sounds and a chronic mottled appearance of her left lower extremity.

A chest radiograph showed a possible left lower lobe consolidation. Follow-up computed tomography scan showed a large bilateral PE with concern for right ventricular (RV) strain. An order for heparin was placed; however, this was never able to be started as 20 min after admission, the patient suffered a cardiac arrest. Return of spontaneous circulation was achieved after 30 seconds of compressions.

**Fig. 1:**
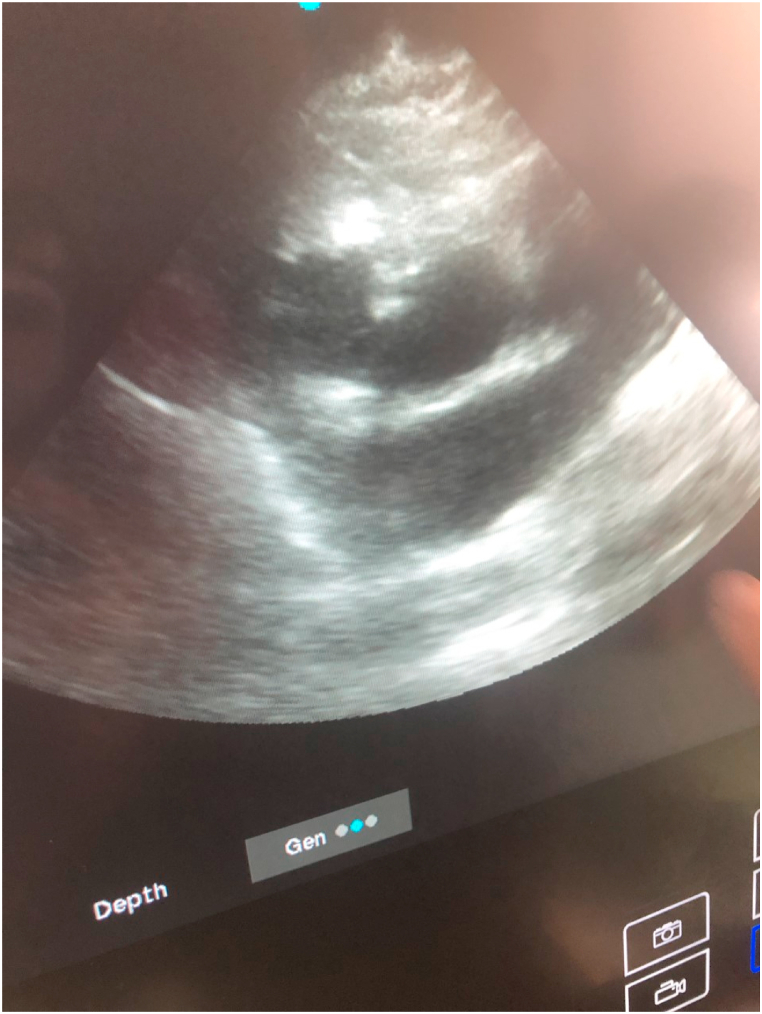
Left atrial mass and right ventricular dysfunction seen on bedside cardiac ultrasound.

Intensive Care evaluation was requested at the time of the cardiac arrest. Upon bedside examination, the patient had an adequate blood pressure of 160's/90'smmHg, with normal sinus heart rate without tachycardia, and saturating well on a nonrebreather mask. Cardiac ultrasound was performed at bedside showing significant RV strain as well as a mobile mass in the left atrium (See Fig. 1). Cardiothoracic surgery was requested who further evaluated the patient at bedside. Due to the ultrasound findings and correlation with the CT findings showing an obstructive effect from the left atrial mass, the patient was emergently taken to the Operating Room (OR) for Pulmonary Thromboendarterectomy (PTE) and left atrial mass excision.

**Fig. 2:**
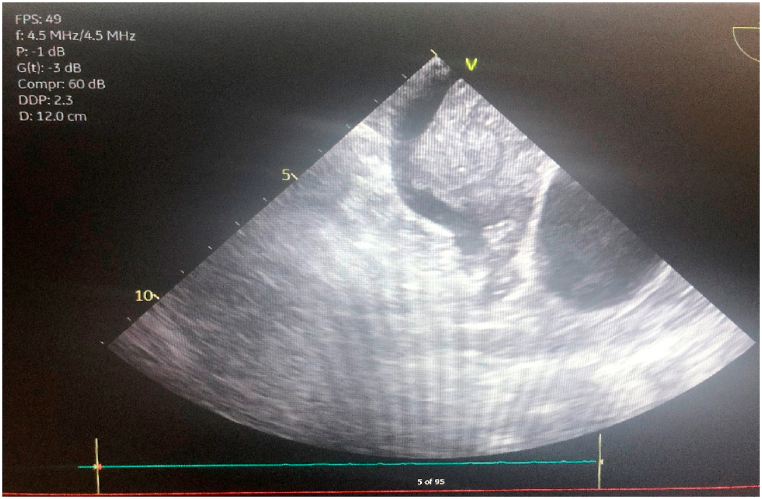
Left atrial mass seen on *trans*-esophageal echocardiogram in the OR.

**Fig. 3:**
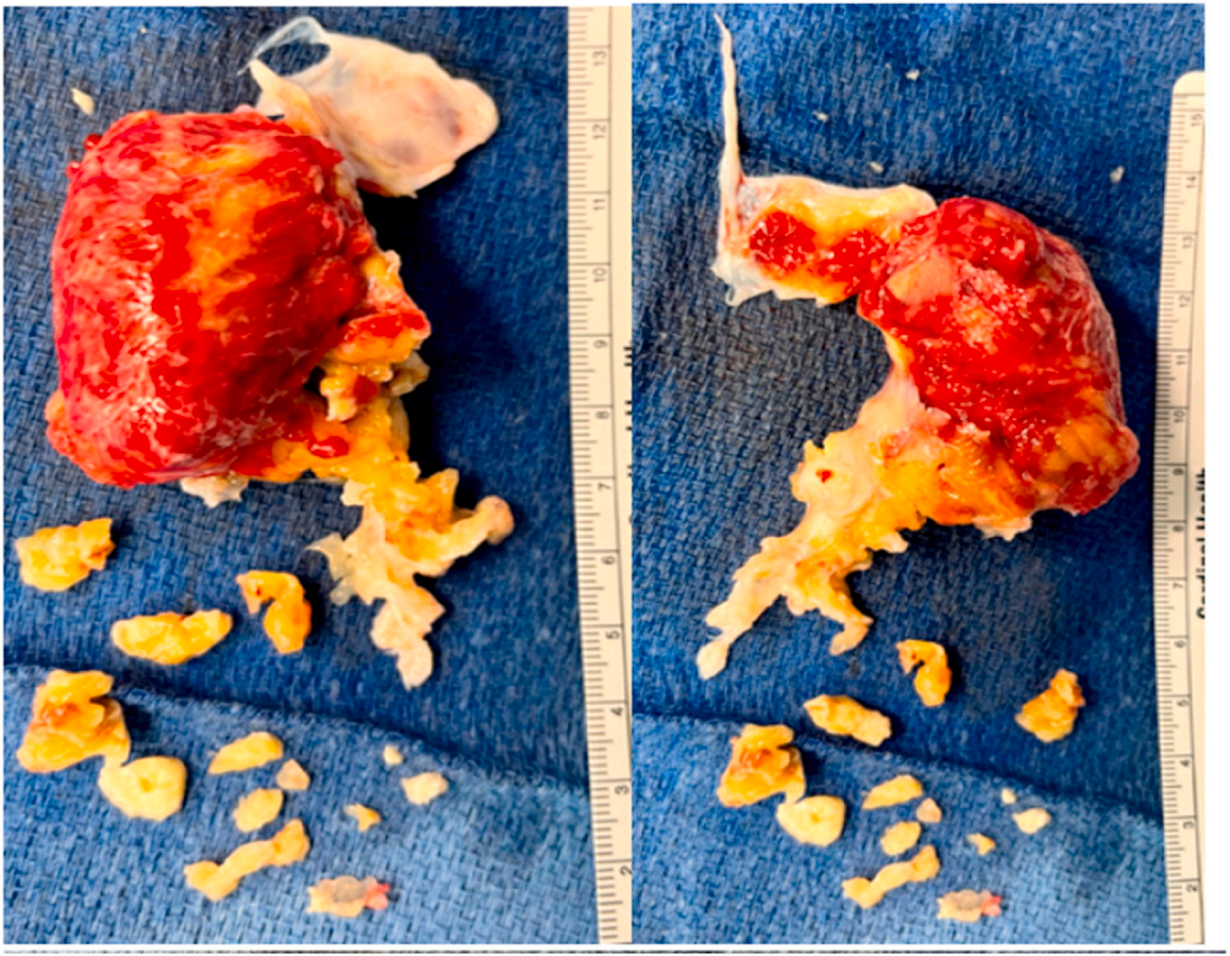
Mass excised from left atrium.

**Fig. 4:**
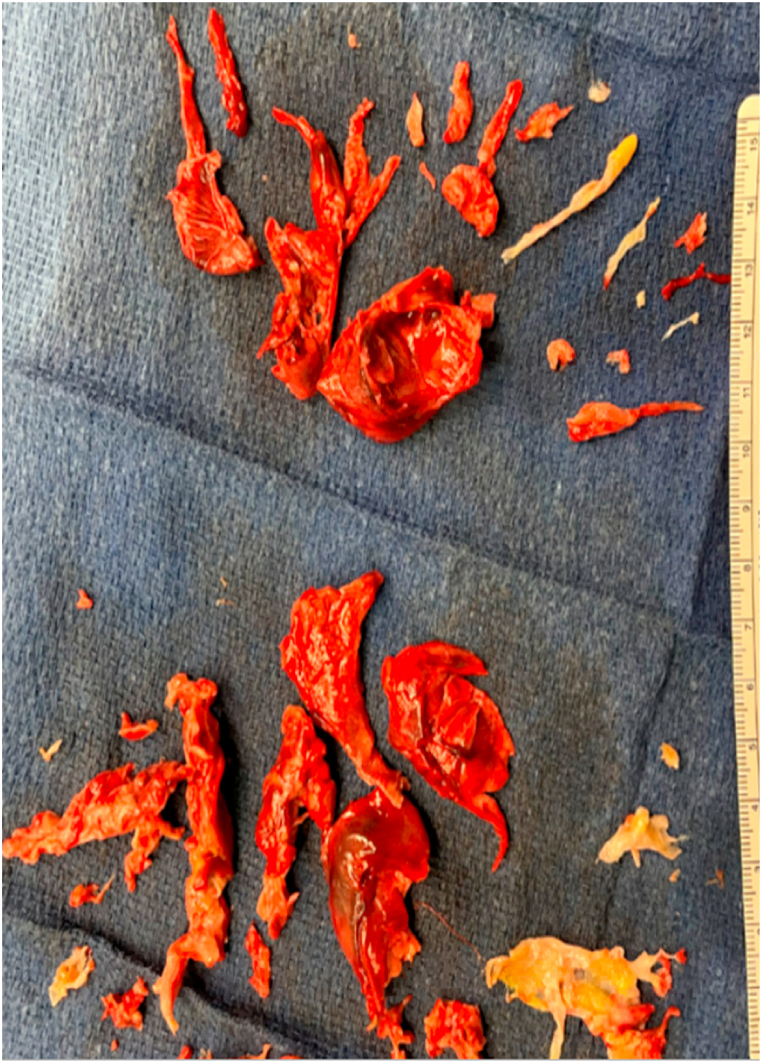
Clot excised from pulmonary arteries.

In the OR, thrombectomy and thromboendarterectomy of the left and right pulmonary arteries was performed. The pulmonary veins were dissected and treated with radiofrequency ablation. Subsequently, the left atrium was opened exposing a large mass originating from the left atrial appendage adherent to the left atrial wall between the left pulmonary veins and the appendage with clots extending to the left superior pulmonary vein. Residual clots were removed and the atrium was closed. The clots were found to have a chronic nature to them, requiring a prolonged deep hypothermic circulatory arrest time. Unfortunately during the procedure, the patient became hypoxic and required initiation of venoarterial extracorporeal membrane oxygenation (VA-ECMO). Additionally, a hemodialysis line was placed in her right femoral vein and the patient was ultimately transferred critically ill to the cardiothoracic intensive care unit (CTICU). Pathology from the extracted material from the pulmonary arteries resulted in a thrombus with a chronic component. Additionally, the left atrial mass pathology showed findings consistent with a myxoma (see Fig. 2, Fig. 3, Fig. 4).

Unfortunately, the patient continued to be hemodynamically unstable with significant lactic acidosis, bloody pulmonary edema and high chest tube output. It was determined she was too unstable to return to the operating room for exploration. After discussion with the patient's family it was decided to transition the patient to a comfort care directed approach. She passed shortly thereafter.

## 3 Discussion

Atrial Myxoma is the most common primary cardiac tumor and is associated with a triad of symptoms including obstruction, emboli and constitutional symptoms [2]. They occur most commonly in the left atrium of females in their fourth and sixth decade of life, which correlates perfectly with our patient. Pulmonary emboli are more frequently associated with right atrial myxoma, while left atrial myxoma are more commonly associated with increased risk of systemic embolization due to the high systolic pressure. Transthoracic echocardiography is the investigational modality of choice [3]. The differential diagnosis for atrial myxoma includes mural thrombi, primary sarcoma, primary cardiac lymphoma, or large b-cell lymphoma. Treatment normally involves surgical excision with the respective tumor sent to pathology to rule out malignancy. If there is successful resection with adequate margins the prognosis for patients with atrial myxoma is excellent - with an operative mortality less than 5%. However, atrial myxomas can reoccur with 22% in complex atrial myxomas, 12% in familial cases and 1–3% in sporadic cases [2].

Right ventricular dysfunction is the defining characteristic of a high-risk intermediate risk PE and is the primary cause of death in pulmonary emboli [4]. Rapid assessment and treatment of intermediate risk PE's remains paramount. Bedside cardiac ultrasounds have become a well-established tool to aid in this rapid assessment [5].

As this patient did have evidence of RV dysfunction without hemodynamic compromise, her PE was categorized as high risk intermediate PE. Her treatment approach could have included anticoagulation alone, low dose tpa, catheter-directed thrombolysis (including EKOS), or surgical thrombectomy. The finding of her left atrial mass directed her care towards a surgical approach as no other option approach would simultaneously eradicate her mass.

In conclusion, intermediate risk pulmonary emboli remains a treatment dilemma requiring a multidisciplinary approach and bedside cardiac ultrasound remains an important tool to help diagnose and risk stratify. While bedside ultrasound is mostly focused on the right side of the heart, we present a case highlighting the importance of looking at every chamber. Atrial myxomas can not only lead to pulmonary emboli, they can also complicate the management of them.

## Declaration of competing interest

We have no conflicts of interest to disclose.

## Appendix A Supplementary data

Video 1 Left atrial mass and right ventricular dysfunction seen on bedside cardiac ultrasound. Video 1
